# Developmental potential of clinically discarded human embryos and associated chromosomal analysis

**DOI:** 10.1038/srep23995

**Published:** 2016-04-05

**Authors:** Guidong Yao, Jiawei Xu, Zhimin Xin, Wenbin Niu, Senlin Shi, Haixia Jin, Wenyan Song, Enyin Wang, Qingling Yang, Lei Chen, Yingpu Sun

**Affiliations:** 1 Center for Reproductive Medicine, the First Affiliated Hospital of Zhengzhou University, Zhengzhou, China

## Abstract

Clinically discarded human embryos, which are generated from both normal and abnormal fertilizations, have the potential of developing into blastocysts. A total of 1,649 discarded human embryos, including zygotes containing normal (2PN) and abnormal (0PN, 1PN, 3PN and ≥4PN) pronuclei and prematurely cleaved embryos (2Cell), were collected for *in vitro* culture to investigate their developmental potential and chromosomal constitution using an SNP array-based chromosomal analysis. We found that blastocyst formation rates were 63.8% (for 2Cell embryos), 22.6% (2PN), 16.7% (0PN), 11.2% (3PN) and 3.6% (1PN). SNP array-based chromosomal analysis of the resultant blastocysts revealed that the percentages of normal chromosomes were 55.2% (2Cell), 60.7% (2PN), 44.4% (0PN) and 47.4% (0PN). Compared with clinical preimplantation genetic diagnosis (PGD) data generated with clinically acceptable embryos, results of the SNP array-based chromosome analysis on blastocysts from clinically discarded embryos showed similar values for the frequency of abnormal chromosome occurrence, aberrant signal classification and chromosomal distribution. The present study is perhaps the first systematic analysis of the developmental potential of clinically discarded embryos and provides a basis for future studies.

Embryo development is a complex process, initiating with the union of the spermatozoon and oocyte and resulting in zygote formation. Clinically, the most important prerequisite for the selection of embryos suitable for transfer is the fertilization of an oocyte by a single spermatozoon prior to zygote formation^1^. However, some embryos can develop from abnormally fertilized zygotes due to the occurrence of polyspermy, which is a common occurrence in human assisted reproductive techniques (ART). This phenomenon results in a zygote with more than two pronuclei. It is estimated that 2–5% of zygotes generated in IVF centers are tripronuclear (3PN) and embryos developed from these zygotes are usually discarded^2^,^3^.

Human embryonic stem cell lines (hESCs) can be derived from the inner cell mass (ICM) of blastocysts and are capable of self-renewal and differentiation into multiple cell lineages representing three embryonic germ layers^4^. The differentiation potential associated with these hESCs has raised hope that they possess the potential for utilization in regenerative medicine. However, the use of embryos and derivation of hESCs for research has polarized ethical debates with much of the contention centered on embryo destruction^5^. Consequently, the generation of new sources of hESCs is important^2^,^6^. During the establishment of cell-based therapies from hESCs, it is necessary to generate numerous cell lines, as each line has its own characteristics and advantages for different applications. Therefore, many researchers have proposed the establishment of a stem cell bank containing hundreds of thousands of ‘immunophenotyped’ stem cell lines encompassing the vast spectrum of transplant antigens, thus avoiding potential immune rejection of hESCs following transplantation^7^. In addition, disease-associated models that use hESCs can also be generated using genetic methods. Using these methods, hESCs can be induced to differentiate into specific cell types, tissues, and organs, which can be used to investigate the mechanisms underlying specific diseases at various stages. Human embryonic stem cells can also be used to screen the clinical efficiency of newly developed drugs.

Because of the associated applications, demand for new hESCs has motivated many researchers to investigate alternative sources for the derivation of hESCs. A major focus of these efforts has involved the study of abnormally fertilized zygotes, which are predominantly discarded^2^,^3^,^8^. Several groups have facilitated the derivation of diploid hESCs from 0PN (without any pronucleus) zygotes^3^, mononuclear (1PN) zygotes^9^ and 3PN zygotes^3^,^8^,^10^. Chen *et al.* reported that 130 3PN zygotes were collected from patients undergoing conventional *in vitro* fertilization (IVF). From these zygotes, 12 blastocysts were formed and four hESCs derived. Three of the associated hESCs demonstrated a normal karyotype^8^. In addition, Suss-Toby *et al.* reported the derivation of normal karyotypical hESCs from 1PN zygotes^9^. In a recent study from our group, hESCs were derived from 3PN zygotes from patients with known diseases^10^.

Single nucleotide polymorphism (SNP) microarray technology has been used in preimplantation genetic screening (PGS)^11^,^12^. Compared with fluorescence *in situ* hybridization (FISH) and comparative genomic hybridization (CGH), SNP microarray technology permits the generation of both qualitative (genotype) and quantitative (gene copy number) data pertaining to entire chromosomes. This technology can also be used to screen a wide variety of genetic disorders^12^,^13^. In ART clinics, SNP microarrays have been applied to genetic analyses of blastomeres from preimplantation embryos^14^,^15^,^16^ and trophectoderm cells from blastocysts^17^ mainly for the detection of aneuploidies of the 46 chromosomes.

In this study, a total of 1,649 clinically discarded embryos were collected on Day 3 after IVF and ICSI, allowed to develop by *in vitro* culture to the blastocyst stage. Starting embryos comprised 0PN, 1PN, 3PN, ≥4PN (at least four pronuclei), 2Cell (prematurely cleaved at the time of pronuclear checking) and poor quality embryos derived from 2PN. The chromosomal constitution of blastocysts and arrested embryos were assessed by SNP microarray analysis. This is perhaps the first study to systematically evaluate the developmental potential of clinically discarded embryos and their genetic status using SNP microarray-based 24 chromosome aneuploidy screening. This work provides the basis for the future utilization of clinically discarded embryos in clinical and basic research.

## Materials and Methods

### Human embryo samples

All embryos were used after receiving written informed consent from couples at the Center for Reproductive Medicine in the First Affiliated Hospital of Zhengzhou University. The study was approved by the Biomedical Ethics Committee at the First Affiliated Hospital of Zhengzhou University and was conducted in compliance with the Declaration of Helsinki for Medical Research.

### Ovum pick-up and fertilization

Cumulus-oocyte-complexes (COCs) retrieval was performed at 36–37 h post-hCG injection. These were subsequently cultured for 2–3 h in G-IVF medium (Vitrolife Sweden AB, Goteborg, Sweden) supplemented with 10% HSA solution (Vitrolife) at 37 °C with 6% CO_2_, to facilitate further maturation. Spermatozoa were selected using a two-layer Spermgrad (Vitrolife) gradient (90% and 45%) and/or swimming up methods. *In vitro* fertilization (IVF) was performed by incubating each oocyte with 10,000 spermatozoa in a 50 μl fertilization microdrop. Prior to intracytoplasmic sperm injection (ICSI), COCs were denuded using hyaluronidase (80 U) and then cultured further for 1–2 h.

### Pronuclear checking and Embryo culture

Pronuclear checking was performed using an inverted microscope (TE2000-U, Nikon, Japan) at 19–20 h post-fertilization. Concomitantly, the numbers of pronuclear and cleaved embryos were recorded.

The cleavage staged embryos were singularly cultured in 50 μl microdrops containing G1 medium (Vitrolife) supplemented with 5% HSA (Vitrolife), at 37 °C and 6% CO_2_. On the morning of Day 3 of culture, embryos were observed using an inverted microscope and scored according to the Peter cleavage-stage embryo Scoring system^18^. Abnormal embryos following fertilization, such as those with no obvious pronucleus (0PN), one pronucleus (1PN), three pronuclei or higher (3PN and ≥4PN) and prematurely cleaved embryos (2Cell), were collected for further culture *in vitro*. In addition, poor quality embryos developed from normal fertilization (2PN) on Day 3, such as embryos with fewer than five blastomeres or with higher than 50% fragments, were also collected as part of this study (Supplementary Figure S1).

### Blastocyst culture and biopsy

Collected embryos were cultured singularly at 37 °C and 6% CO_2_ in 50 μl microdrops containing G2 medium (Vitrolife) supplemented with 6% HSA. On the morning of Day 5 or 6 of culture, embryos were examined using an inverted microscope and scored according to the Gardner blastocyst Scoring system^19^ (Supplementary Figure S2). High-quality blastocysts represent those with grades equal or higher than 3BB (Day 5) or 4BB (Day 6). Only those with obvious blastocoel cavities were biopsied using a micromanipulation system (Narashige, Japan) that had been fitted onto the inverted microscope (Nikon). This approach allowed us to distinguish trophoblast cells. After laser-assisted zona drilling (ZILOS-tk, Hamilton Thorne), 3–5 trophoblast cells (TE) from Day 5 or Day 6 blastocysts were retrieved for SNP analysis using biopsy needles. For non-blastocysts, 3–5 cells with clear cell morphologies were retrieved using biopsy needles after laser-assisted zona drilling. These cells were also subjected to SNP analysis.

In addition, a total of 111 blastocyst-related SNP results from 54 couples were collected from the clinical PGD samples that were used in this study. The biopsy procedure used to retrieve cells from PGD samples was consistent with that used for clinically discarded embryos.

### SNP microarray analysis

Biopsied cells were collected using 135 μm diameter needles (Origio, Måløv, Denmark). The cells were subsequently lysed in 5 μl of cell lysis buffer (0.2 M KOH). The samples were subjected to DNA denaturation and whole genome amplification (WGA) using a REPLI-g Midi MDA Kit (Qiagen, Düsseldorf, Germany). The total lysate was amplified in a 40 μl reaction mix at 30 °C for 4 h, and the reaction was terminated following a three-minute incubation step at 65 °C. Four microliters (approximately 200 ng) of each MDA product was subjected to denaturation using an alkaline denaturation buffer (0.1 M NaOH), before amplification was performed at 37 °C for 13 h using the Human CytoSNP-12 DNA analysis beadchips Kit (Illumnia, San Diego, CA).

The amplified DNA product underwent enzymatic end-point fragmentation prior to precipitation and resuspension in the hybridization buffer. Fragmented and resuspended DNA samples were dispensed onto Human CytoSNP-12 DNA analysis beadchips. The samples were subsequently hybridized with the beadchip at 48 °C for 12 h. Following hybridization, the beadchips were subjected to immunostaining followed by stringent washing in order to remove non-specific hybridized and un-hybridized DNA samples.

The beadchips were dried in a vacuum desiccator and scanned using an Illumina iScan Bead Array Reader. Raw data were analyzed using Illumina Genome Studio software and Karyostudio software. Prior to commencement of the study, an embryonic cell normalized data set was established as a reference database. The research data generated as a result of this analysis were compared to the reference data. Data relating to SNPs with poor or incomplete genotype information were removed. Results from the Illumina system were used to evaluate whether each sample was normal (diploid) and/or contained a genomic imbalance or aneuploidy.

### Statistical analysis

Statistical analysis was performed using SPSS13.0 software^20^. The proportional data were compared using Chi-squared analysis or Fishers Exact test, and the significant difference value was set at P < 0.05. The percentage of blastocyst formation represents the number of blastocysts formed, divided by the total number of embryos cultured; the percentage of high-quality blastocyst formation represents the number of high quality blastocysts divided by the total number of blastocysts cultured.

## Results

### Blastocyst formation from discarded embryos derived from different sources

A total of 1,649 discarded embryos from 556 couples were collected for further culture. The average age of females recruited as part of this study was 30.29 ± 5.07 years, while the average age of males was 30.61 ± 5.53 years. All discarded embryos were cultured *in vitro* from zygotes showing different numbers of pronuclei (0PN, 1PN, 2PN, 3PN and 4PN or higher) and premature cleavage (2Cell) at Day 1. A total of 253 blastocysts were formed (15.3%). Among these, the blastocyst formation rate in the 2Cell group was the highest (63.8%, *P* = 0.000). The rate of 2PN-derived embryos (discarded embryos with ≤4 blastomeres and/or fragments higher than 50%) was 22.6%. This result was significantly (*P* = 0.000) higher than results observed for the 1PN, 3PN, and ≥4PN groups. The rate of blastocyst formation was 16.7%, 3.6%, 11.2%, and 3.3%, in 0PN-, 1PN-, 3PN- and ≥4PN-derived discarded embryos, respectively.

**Figure 1:**
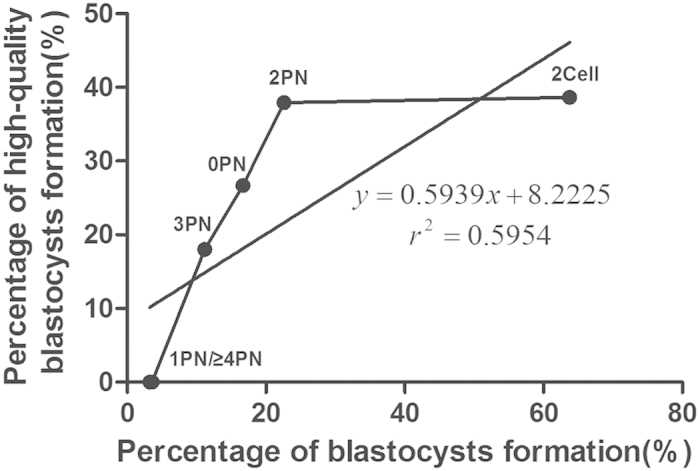
Correlation between percentages of blastocyst formation and high-quality blastocyst formation from different zygotes and prematurely cleaved 2Cell embryos. Percentages are of blastocyst formation (x-axis) and high-quality blastocyst formation (y-axis).

Some high-quality blastocysts were obtained despite the use of discarded zygotes or 2Cell embryos. The high-quality blastocysts represent those with grades equal or higher than 3BB (Day 5) or 4BB (Day 6). Indeed, the total high-quality blastocyst formation rate was 27.7%. Among these, the percentages of high-quality blastocysts in the 2Cell and 2PN groups were 38.6% and 37.9%, respectively. Additionally, 26.7% and 18.0% of 0PN and 3PN zygotes developed into blastocysts, respectively. The correlation between the percentage of blastocyst formation from embryos derived from different discarded embryos and the percentage of high-quality blastocyst formation was analyzed, and the results showed that there was a significant positive linear correlation between the two groups (*P* = 0.036, one-tailed *t*-test) (Fig. 1). These results indicated a correlation between blastocyst and high-quality blastocyst formation rates (Table 1).

The results of the analysis of the detailed age groups of oocyte donor and sperm donor showed that the young oocyte donor have higher embryo developmental potential (Supplementary Tables S1 and S2).

### SNP array-based chromosome analysis of discarded embryos from various sources

Blastocysts and non-blastocysts that developed from the 0PN, 2PN, 3PN, and 2Cell embryos were used for SNP microarray analyses to explore the high percentage of blastocyst formation further (Table 2). Both normal and abnormal chromosomes were present following embryo development. A total of 235 SNP microarray results were generated and the results showed that there was no significant difference between the percentage of normal and abnormal chromosome SNP microarray signals (53.6% vs 46.4%, *P* = 0.104), regardless of the original embryo type. In the blastocyst formation group, there were no significant differences in the normal signal rate among the 0PN, 2PN, 3PN and 2Cell groups (*P* = 0.357). However, for embryos unable to form blastocysts (non-blastocysts), the percentage of normal chromosome signal in 2PN-derived embryos was significantly higher than in 3PN-derived embryos (75.0% vs 22.7%, *P* = 0.0001).

### Classification of different SNP array-based chromosomal aberration signals

**Figure 2:**
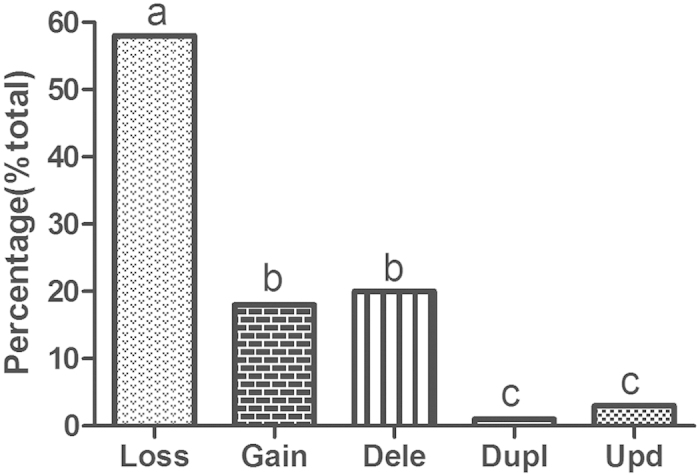
Analysis of abnormal SNP array signals. Frequently-detected abnormal signals following SNP array-based chromosome analysis were analyzed by dividing the number of distinct signals by the number of abnormal signals. “Loss”, lack of an entire chromosome; “Gain”, an increase in the number of copies of a chromosome; “Dele”, segmental chromosome deletion; “Dupl”, segmental chromosome duplication; “Upd”, uniparental disomy. Different alphabets on the histogram indicate significant differences (*P* < 0.05).

Various chromosomal abnormalities, including whole chromosome loss and gain, segmental chromosome deletion and duplication, and uniparental disomy (UPD) were also investigated (Fig. 2). Chromosome loss was the most frequently-occurring chromosome abnormality (58.2%) (*P* = 0.000). Both chromosome gain and segmental chromosome deletion were observed more frequently than segmental chromosome duplication and UPD (17.6%, 20.5% vs 0.8%, 2.9%; *P* = 0.0005) but there were no significant differences among chromosome gain and segmental chromosome deletion (*P* = 0.72), or segmental chromosome duplication and UPD (*P* = 0.31).

### Distribution of SNP array-based chromosome aberration signals from different sources of discarded embryos

A total of 244 abnormal chromosome signals were categorized according to chromosomal locus. The most frequent abnormal signals were localized to chromosomes 8, 9, 16 and the sex chromosomes, whereas chromosomes 1, 6, 15 and 18 exhibited fewer abnormal signals (*P* = 0.024; Supplementary Figure S3).

In addition, the total number of abnormal signals and the different abnormal signal types, including whole chromosome loss and gain, and segmental chromosome deletion, were analyzed in blastocysts derived from discarded embryos (Supplementary Figures S4–S13). In the 0PN group, the occurrence of abnormal signals was localized to chromosomes 9 and 11 (Supplementary Figure S4), with chromosome loss and segmental chromosome deletion representing the predominant source of aberrant signals (Supplementary Figure S5). In the 2PN group, chromosomes 4, 8, 10 and 16 resulted in the generation of the highest proportion of abnormal signals (Supplementary Figure S6). Chromosome abnormalities such as loss and deletion were also high in this group (Supplementary Figure S7). Chromosomes 5, 9, 12, 16, 22, and the sex chromosomes had the highest number of abnormal signals in the 3PN group (Supplementary Figure S8), with whole chromosome loss representing the most common abnormality. Interestingly, chromosome gain was frequently associated with the sex chromosomes. In addition, uniparental disomy was restricted to the 3PN group (Supplementary Figure S9). In embryos arising from the 2Cell group, abnormal signals were mainly localized to chromosomes 9, 16, 17, 20 and the sex chromosomes (Supplementary Figure S10). Furthermore, whole chromosome loss and segmental chromosome deletion were the predominant abnormalities (Supplementary Figure S11).

### Comparison of blastocysts from PGD samples and discarded embryos using SNP array-based chromosome analysis

To investigate differences associated with chromosomal abnormalities between blastocysts derived from discarded embryos and those used for clinical preimplantation genetic diagnosis (PGD), we used PGD samples from trophoblasts where implantation had repeatedly failed or from couples with balanced translocation. We collected a total of 111 blastocyst-related SNP results from 54 couples from clinical PGD samples. The average patient age was 31.00 ± 4.92 years for females and 32.30 ± 6.60 years for males. Comparison of SNP array-based chromosome analysis data from PGD and discarded embryos showed that there was no significant difference in the percentage of normal chromosome signals (*P* = 0.369) among blastocysts from PGD and clinical discarded embryos, which were 59.5% (for the PGD group), 44.4% (0PN), 60.7% (2PN), 47.4% (3PN) and 2Cell (55.2%).

**Figure 3:**
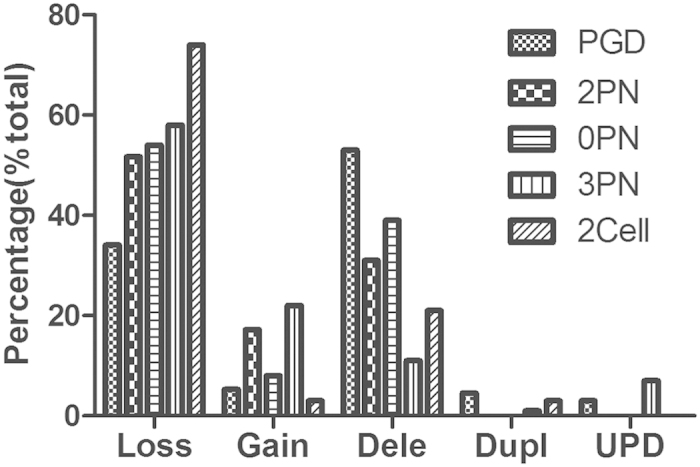
SNP array-based chromosome analysis of blastocysts from clinical PGD samples and discarded embryos. Frequently-detected abnormal signals following SNP microarray analysis were analyzed by dividing the number of distinct signals by the number of abnormal signals. “PGD” refers to data from PGD patients. These data were compared with data generated from blastocysts derived from 2PN-, 0PN-, 3PN-, and 2Cell-derived embryos; other abbreviations are as for Fig. 2.

Abnormal chromosome signals in the clinical PGD samples occurred frequently at chromosomes 1, 2, 7, 14, 20 and 22 (Supplementary Figure S12) with chromosome loss and segmental chromosome deletion contributing most frequently (Supplementary Figure S13). These results demonstrate that blastocysts from clinical PGD samples and discarded embryos share similar frequencies of chromosome anomalies as revealed by whole chromosomal loss and gain, segmental chromosome deletion and duplication, and UPD (Fig. 3).

## Discussion

During *in vitro* fertilization-embryo transfer (IVF-ET), a large number of human gametes and embryos are discarded. In this study, we characterized a total of 1,649 Day 3 embryos that had developed from zygotes of abnormally fertilized (0PN, 1PN, 3PN, and ≥4PN) embryos, poor quality normally fertilized embryos (2PN), and 2Cell embryos that had prematurely-cleaved at the time of pronuclear checking. The embryos were collected and cultured further *in vitro* to facilitate the development of blastocysts. The results reveal that some of these clinically discarded embryos have the potential to develop into blastocysts: 15.3% and 27.7% reached blastocyst and high quality blastocyst stages respectively. The high-quality blastocysts represent those with grades equal or higher than 3BB (Day 5) or 4BB (Day 6). Considering this developmental potential associated with all groups of the discarded embryos, embryos like them could potentially be used as a resource for future investigations.

Previous reports demonstrated that approximately 1% of human zygotes without observable pronuclei but have two polar bodies^21^. A retrospective analysis performed on the transfer of 0PN-derived fresh cleavage-stage embryos, frozen-thawed cleavage stage embryos and frozen-thawed blastocyst-stage embryos, showed that all three groups had the potential to result in pregnancies and live births^22^. However, the authors also found that the pregnancy rate of 0PN-derived cleavage-stage embryos was lower than that of 2PN-derived cleavage stage embryos. The pregnancy rate of blastocyst-stage embryos was comparable with that of 2PN-derived blastocysts^22^. In order to ensure safety, we attempted to culture 0PN-derived embryos, which were abnormally fertilized or normally fertilized but missing both pronuclei^3^, to the blastocyst stages before using the derived blastocysts that contained normal chromosomes (following trophoblast cell biopsy) for genetic analysis, such as SNP microarray analysis. In our study, only 44.4% of blastocysts derived from the 0PN group, where 9 blastocysts were analyzed, possessed a normal chromosomal composition. Thus, the safety of the transfer of 0PN-derived blastocysts merits further analysis.

Consistent with our results, a retrospective study indicated that the percentage of blastocyst formation from 1PN-derived zygotes was significantly lower than that from 2PN-derived zygotes (*P* = 0.000)^23^. Liao *et al.* reported that the blastocyst formation rate from 1PN-derived zygotes was 26.9% (62/231)^24^, which was significantly higher than that observed in this analysis (3.6%, *P* = 0.0067). The differences in the rate of blastocyst formation from 1PN zygotes observed in our study and others may arise from the limited data generated from our samples. However, it is noteworthy that previous reports showed successful pregnancy following transfer of 1PN-derived embryos^23^,^25^. Nine normal healthy babies were born from 33 blastocysts derived from 1PN zygotes transferred following regular IVF. Similar results were not observed following ICSI cycles^23^. Several studies have been shown that the diploid chromosome constitution from IVF derived 1PN zygotes were higher than that derived from ICSI^26^,^27^,^28^. In fact, 1PN zygotes following regular IVF, are mainly formed by the exclosure of the juxtaposed male and female pronuclei within a common pronuclear evelope^29^. However, most of the 1PN zygotes following ICSI are parthenogenesis^30^.

We found that 2PN-derived poor-quality embryos, including slow-cleaving and predominantly fragmented embryos were also able to develop. In a previous study, 14.6% of 130 2PN-derived poor quality Day 3 embryos formed blastocysts^31^. Blastocyst formation was in proportion to the number of blastomeres present, with a 14.3% formation rate being observed when 5–6 blastomeres were present and a 7.4% formation rate when less than five blastomeres were observed in Day 3 embryos^32^. These rates were lower than those observed in our study, with differences possibly due to culture media, manipulation and embryo scoring criteria. At present, the commercial *in vitro* fertilization medium is in constant upgrading to better improve the successful rate of IVF-ET cycles, and culture medium from different manufacturers are different in the rate of blastocyst formation. The outcome of IVF can be affected by different proficiency in the operation, different operation processes and operation skills. In addition, both the use of different embryo scoring criteria and the subjective factor from the embryologist may lead to the difference in the prediction of the development potential of the embryo. Differences aside, this research demonstrates that hESCs can be generated from poor quality 2PN-derived embryos^33^,^34^,^35^.

Polyspermy, especially dispermy, is common in IVF, which was also included in this study, occurring in up to 30% of IVF cases^36^. Polyspermy may be caused by many factors including excessive sperm concentration, defective zona pellucida, inadequate oocyte maturity, oocyte ageing and the timing of hCG injection to facilitate oocyte retrieval^3^,^36^. Additional studies have shown that approximately 10% of 3PN zygotes reach the blastocyst stage^2^,^37^. These results are consistent with our observations. In another report, 12 out of 130 3PN zygotes developed into blastocysts (9.2%) and 30 out of 139 poor-quality Day 3 embryos developed into blastocysts (21.6%), with blastocyst formation from 3PN-derived embryos lower than those from 2PN-derived embryos^8^. These results were also consistent with our findings. Also, it has previously been reported that hESCs can be derived from polypronucleated embryos^38^.

Due to the abnormal chromosomal status of abnormally fertilized zygotes, developmental competence and embryo quality can be impaired, resulting in the occurrence of cell fragments or cell apoptosis^2^. These observations are consistent with results generated as part of this analysis, where blastocyst formation rates, high-quality blastocyst rates and the percentage of normal chromosomal occurrence in non-blastocyst groups were all significantly higher in the 2PN group than the 3PN group (*P* = 0.0003). However, there was no significant difference in the percentage of normal chromosomal occurrence in the blastocyst formation between the 2PN and 3PN group (*P* = 0.121). Therefore, we speculate that ‘normalization’ or ‘self-correction’, by which we mean the apparent repair of chromosomal fragmentation, may play an important role in embryonic development^13^,^39^. Interestingly, embryos demonstrate mosaicism, meaning that biopsies on embryos may not be fully representative when it comes to chromosomal composition of the embryo as a whole. Indeed, euploid cells that were not initially sampled during biopsy may come to predominate during the development process, leading to a correction in the chromosomal composition of the embryo. In order to combat this, biopsies performed during this analysis were carried out on blastocysts as opposed to earlier-stage embryos.

A recent report indicated that 57% of 0PN zygotes were euploid, 30% displayed polyploidy or mosaicism, whereas the aneuploidy rate was 13%^40^. In embryos that had developed from 1PN zygotes, diploid rates were 48.7% following conventional IVF and 27.9% following ICSI^41^. We also found that the normal chromosome rates of blastocysts derived from 0PN zygotes were 44.4%, thus suggesting correction of chromosome aneuploidy during embryo culture. Therefore, when 2PN embryos are not available for transfer, 0PN or 1PN embryos could be used following PGD or PGS.

Using an SNP array for clinically discarded embryos, we found that whole chromosome loss is the most frequently occurring abnormal event, consistent with a previous report^42^. In addition, whole chromosome gain and segmental deletions were also frequently observed as previously reported^43^,^44^,^45^. However, UPD, which is rarely observed in human 2PN-derived blastocysts, was frequently detected in human clinically discarded embryos^46^,^47^, consistent with observations made during our analysis. It is possible that genetic diseases occur during embryo development as a result of UPD in specific chromosomes containing imprinted genes^48^.

Following the use of SNP array-based 24 chromosome aneuploidy screening, two out of 3,401 blastocysts demonstrated isodisomy in human 2PN-derived blastocysts after trophoblast cell biopsy, with an associated frequency of UPD of 0.06%^42^. In our previous study, the percentage of UPD in human clinically discarded blastocysts was 3.7%^46^, consistent with data generated as part of the present study (2.7%). These results also indicate that UPD may be a common phenomenon in discarded human embryos, especially in blastocysts derived from abnormally fertilized zygotes. Therefore, we speculate that abnormally fertilized embryos can use UPD to ‘self-correct’ during development^46^.

We found that 0PN, 2PN and 2Cell embryos gave rise to embryos with some similarities, including the occurrence of whole chromosome loss and segmental chromosome deletion. However, whole chromosome loss and gain are more commonly observed in 3PN- and ≥4PN-derived embryos, frequently involving the gain of a sex chromosome. These findings suggest that non-visible pronuclei in embryos derived from 0PN and prematurely-cleaved 2Cell embryos may have two sets of chromosomes (similar to 2PN-derived embryos). However, these pronuclei are different from those derived from embryos with visibly three or more three pronuclei. In addition, embryos derived from 2PN and 2Cell generate a higher percentage of normal chromosome signals compared with other derivations.

By analyzing abnormal SNP array signals in specific chromosomes, we found that aneuploidy more frequently involves certain chromosomes, consistent with some previous reports^49^,^50^, but not others^11^,^39^. This discrepancy may arise from the sensitivity of the techniques used, analysis methods, embryo selection, and sample sizes utilized.

In conclusion, we found that clinically discarded human embryos derived from abnormally fertilized zygotes or normally fertilized zygotes of poor quality can develop to blastocysts. These blastocysts demonstrate different developmental potential. Some could be used for the derivation of hESCs for both clinical and basic research. Based on SNP array analyses, the highest frequency of occurrence of normal chromosome composition was found in 2PN-derived blastocysts. This is perhaps the first systematic study to analyze the developmental potential of clinically discarded embryos and associated chromosome composition. The results provide a basis for the future use of similar embryos for research and clinical applications.

## Additional Information

**How to cite this article**: Yao, G. *et al.* Developmental potential of clinically discarded human embryos and associated chromosomal analysis. *Sci. Rep.* **6**, 23995; doi: 10.1038/srep23995 (2016).

## Supplementary Material

Supplementary Information

## Figures and Tables

**Table 1:** Blastocyst formation from discarded embryos cultured *in vitro.*

Groups	Female age range	Male age range	No. of embryos cultured	No. of blastocysts formed (% embryos)	No. of high-quality blastocysts formed (% blastocysts)
0PN	22–42	23–43	90	15 (16.7%)^abc^	4 (26.7%)^a^
1PN	21–43	22–40	28	1 (3.6%)^bd^	0
2PN	21–42	22–45	385	87 (22.6%)^ae^	33 (37.9%)^a^
3PN	21–43	22–44	893	100 (11.2%)^bf^	18 (18.0%)^b^
≥4PN	21–41	23–40	184	6 (3.3%)^dg^	0
2Cell	22–43	24–42	69	44 (63.8%)^h^	17 (38.6%)^a^
Total			1649	253 (15.3%)	70 (27.7%)

Note: Different superscripts in the same column indicate significant differences (P < 0.05). High-quality blastocysts represent those with grades equal or higher than 3BB (Day 5) or 4BB (Day 6).

**Table 2:** SNP microarray signals from blastocysts and non-blastocysts.

Group	Blastocysts		Non-blastocysts	
	No. tested	No. of normal chromosomes (% total)	No. tested	No. of normal chromosomes (% total)
0PN	9	4 (44.4%)^a^	–	–
2PN	61	37 (60.7%)^a^	36	27 (75.0%)^a^
3PN	78	37 (47.4%)^a^	22	5 (22.7%)^b^
2Cell	29	16 (55.2%)^a^	–	–
Total	177	94 (53.1%)	58	32 (55.2%)

Note: SNP microarray signals from blastocysts and non-blastocysts were subdivided into groups of 0PN, 2PN, 3PN, and premature cleaved 2Cell. Different superscripts in the same column indicate significant differences (P < 0.05), and the same superscripts indicate no significant difference (P > 0.05).
