# Supplementary material for: Developmental potential of clinically discarded human embryos and associated chromosomal analysis
Source: Sci Rep. 2016 Apr 5;6:23995. doi: 10.1038/srep23995 (PMC4820740; doi:10.1038/srep23995)
Supplement: Supplementary Information [file srep23995-s1.pdf]

Developmental potential of clinically discarded human embryos and associated chromosomal analysis

Guidong Yao#, Jiawei Xu#, Zhimin Xin#, Wenbin Niu, Senlin Shi, Haixia Jin, Wenyan Song, Enyin Wang, Qingling Yang, Lei Chen &Yingpu Sun*

# These authors contributed equally to this work

*Address all correspondence to Yingpu Sun, Center for Reproductive Medicine, The first Affiliated Hospital of Zhengzhou University, Zhengzhou 450052, China. Tel.: 86-371-67966166; Fax: 86-371-66913635; E-mail: syp2008@vip.sina.com

Supplementary materials

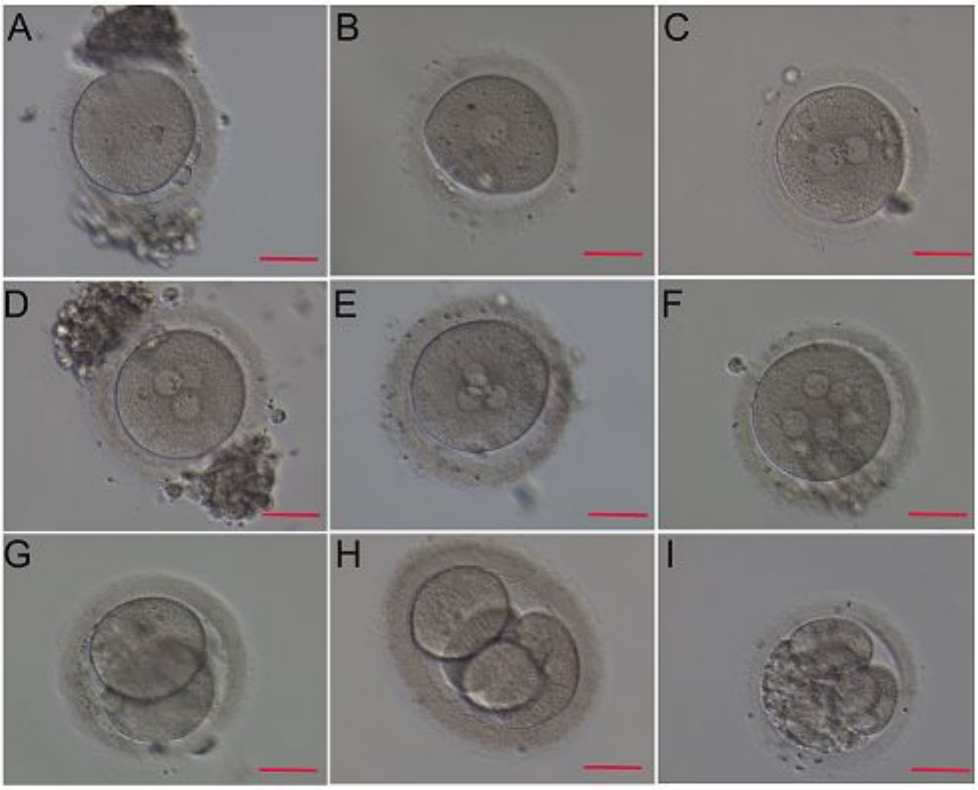

Supplementary Figure S1. Human clinically discarded derived from different sources.

(A-F) 0PN, 1PN, 2PN, 3PN, 4PN and 5PN zygote generated 19-20 h after in vitro fertilization. (G) 2Cell embryo generated 19-20 h after in vitro fertilization. (H) Embryo from 2PN derived zygote with less than 5 blastomeres on Day 3 (slow cleaving). (I) Embryo with more than 50% fragments on Day 3. Bar = 50μm.

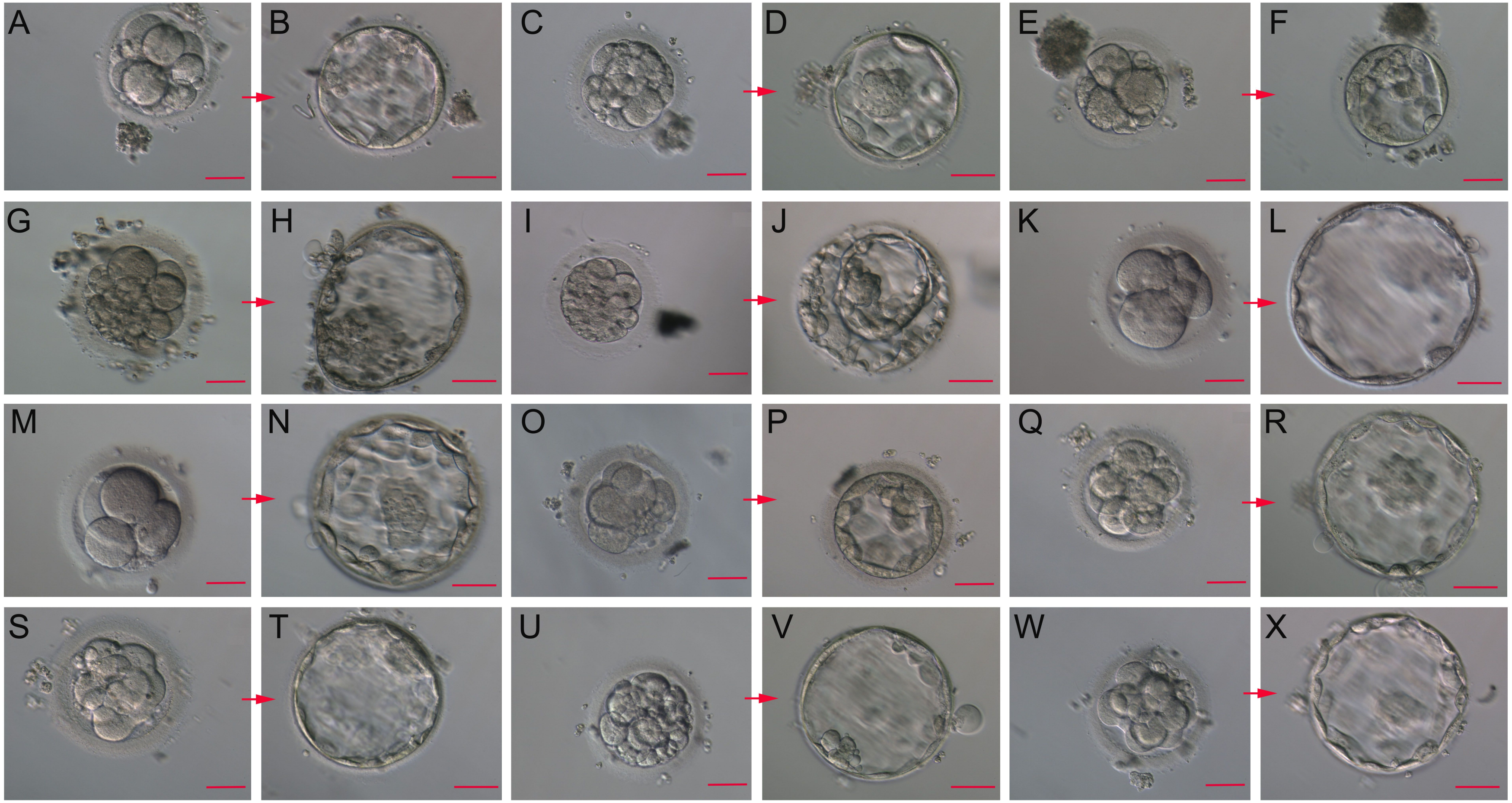
A
B
C
D
E
F
G
H
I
J
K
L
M
N
O
P
Q
R
S
T
U
V
W
X

Supplementary Figure S2. Blastocysts developed from day 3 embryos derived from different sources

(A) Day 3 embryo derived from 0PN and its Day 6 blastocyst (B); (C) Day 3 embryo derived from 0PN and its Day 5 blastocyst (D); (E) Day 3 embryo derived from 1PN and its Day 6 blastocyst (F); (G) Day 3 embryo with fragments derived from 2PN and its Day 6 blastocyst (H); (I) Day 3 embryo with fragments derived from 2PN and its Day 5 blastocyst (J); (K) Day 3 embryo with less than 5 blastomeres derived from 2PN and its Day 6 blastocyst (L); (M) Day 3 embryo with less than 5 blastomeres derived from 2PN and its Day 5 blastocyst (N); (O) Day 3 embryo derived from 3PN and its Day 5 blastocyst (P); (Q) Day 3 embryo derived from 3PN and its Day 6 blastocyst (R); (S) Day 3 embryo derived from 4PN and its Day 6 blastocyst (T); (U) Day 3 embryo derived from 2Cell and its Day 6 blastocyst (V); (W) Day 3 embryo derived from 2Cell and its Day 5 blastocyst (X). Bar = 50μm.

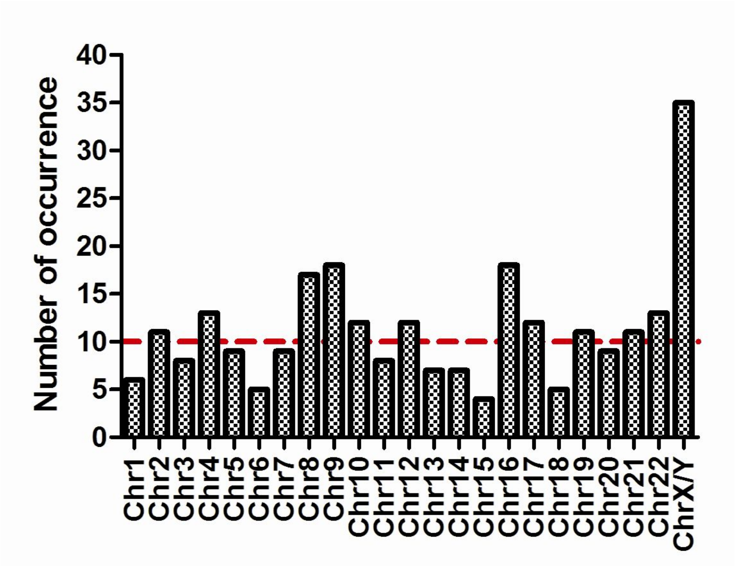

Supplementary Figure S3. Chromosome distribution of SNP array-based aberration signals

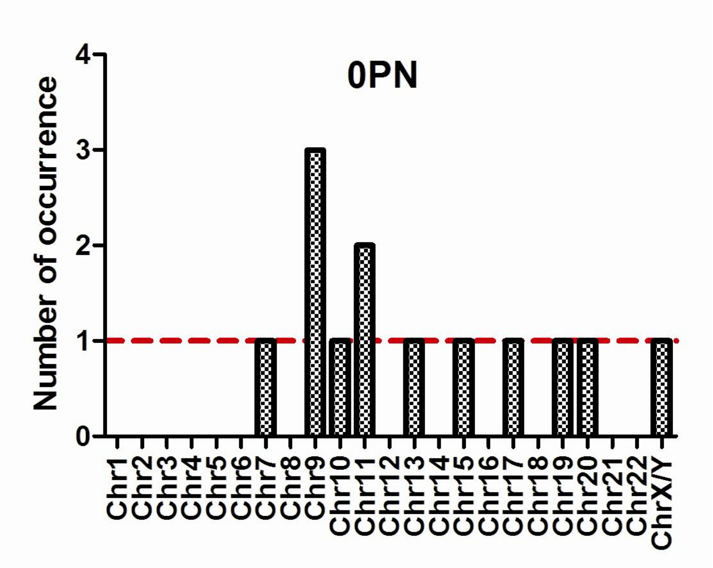

Supplementary Figure S4. Chromosome distribution of SNP array-based aberration signals from 0PN-derived embryos

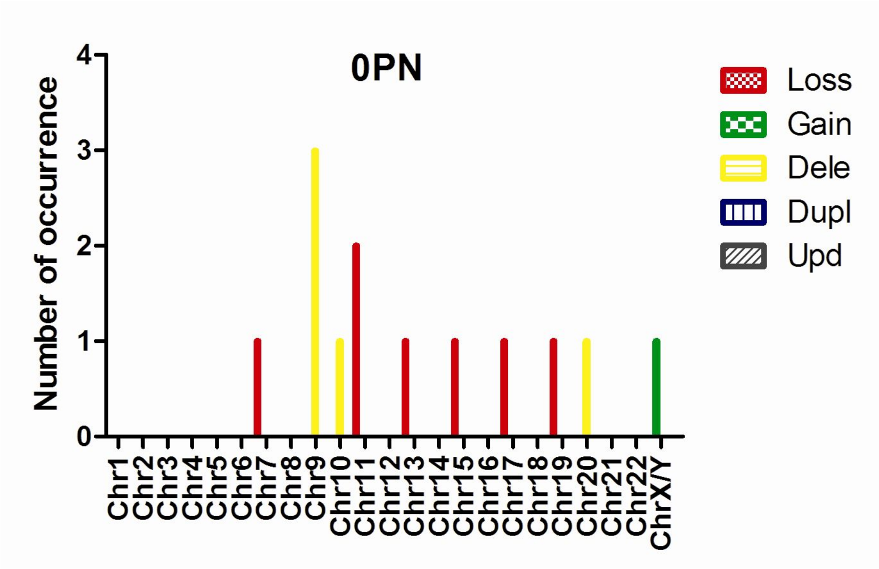

Supplementary Figure S5. Chromosome distribution of SNP array-based different abnormal signals from 0PN-derived embryos

Note: Whole chromosome loss was marked as red (Loss), whole chromosome gain was marked as green (Gain), segmental chromosome deletion was marked as yellow (Dele), segmental chromosome duplication was marked as blue (Dupl), and uniparental disomy (Upd).

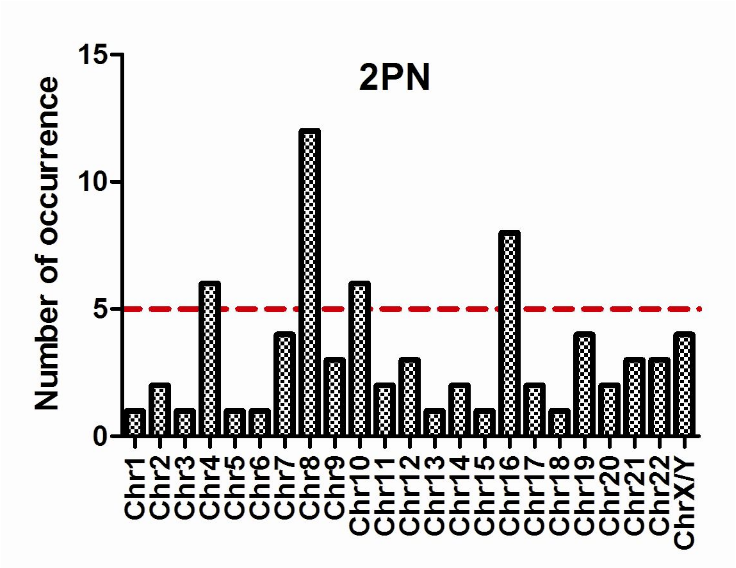

Supplementary Figure S6. Chromosome distribution of SNP array-based aberration signals from 2PN-derived embryos

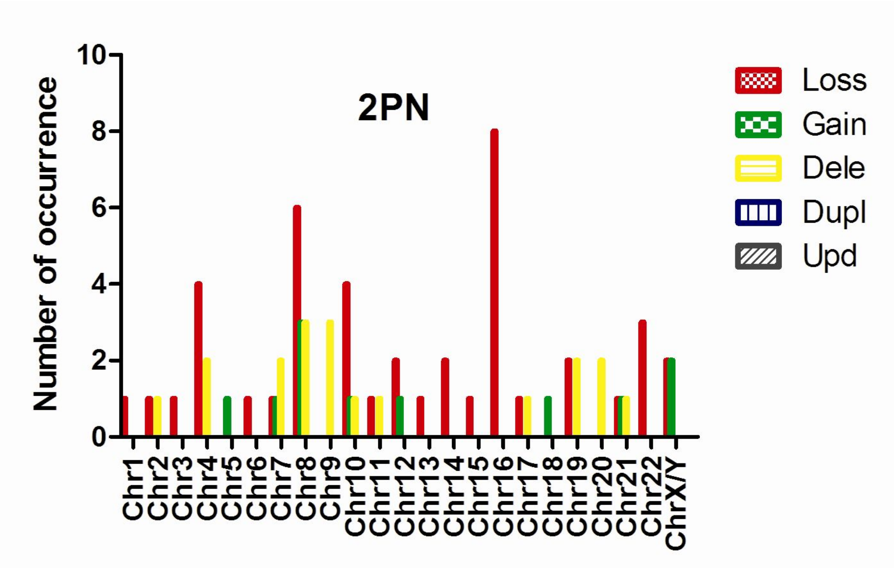

Supplementary Figure S7. Chromosome distribution of SNP array-based different abnormal signals from 2PN-derived embryos

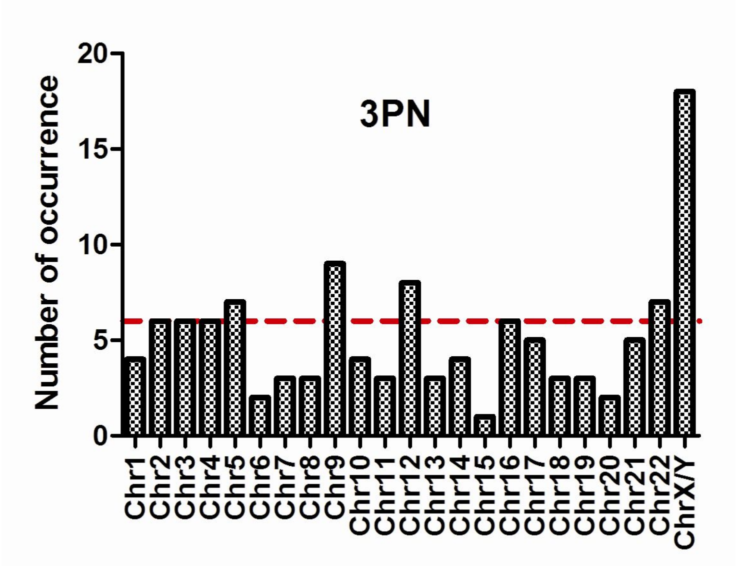

Supplementary Figure S8. Chromosome distribution of SNP array-based aberration signals from 3PN-derived embryos

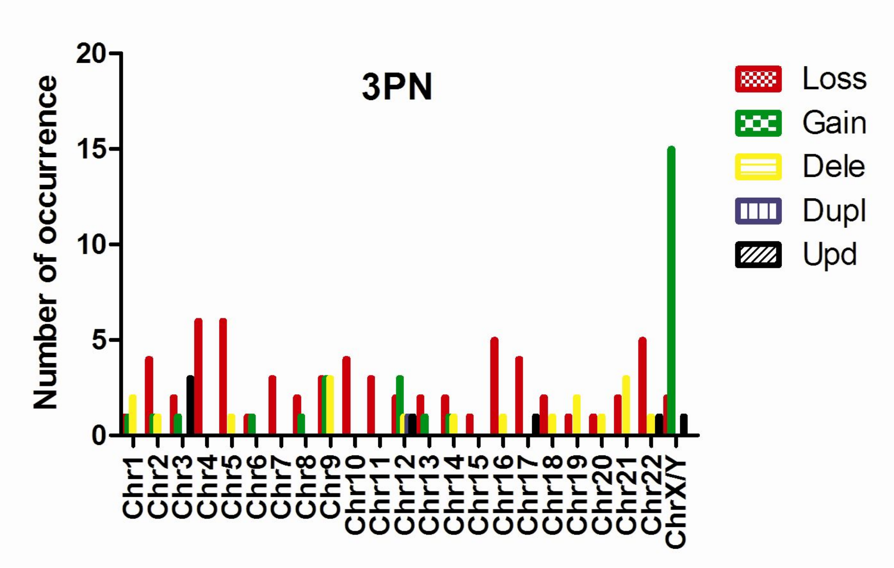

Supplementary Figure S9. Chromosome distribution of SNP array-based different abnormal signals from 3PN-derived embryos

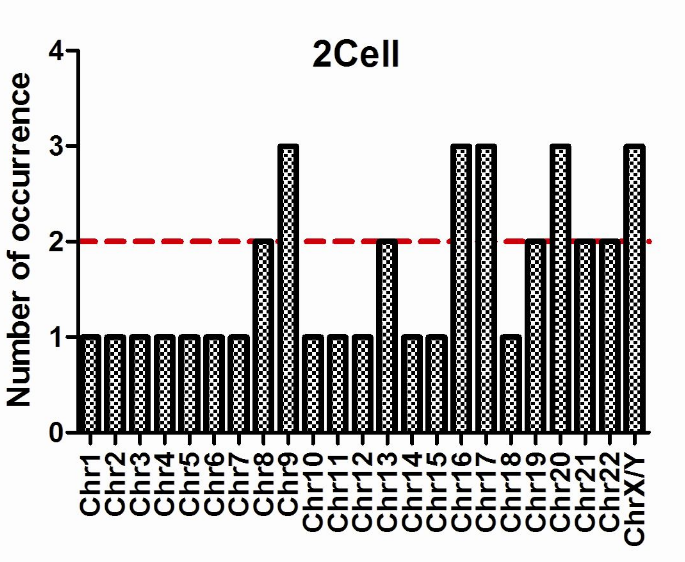

Supplementary Figure S10. Chromosome distribution of SNP array-based aberration signals from 2Cell-derived embryos

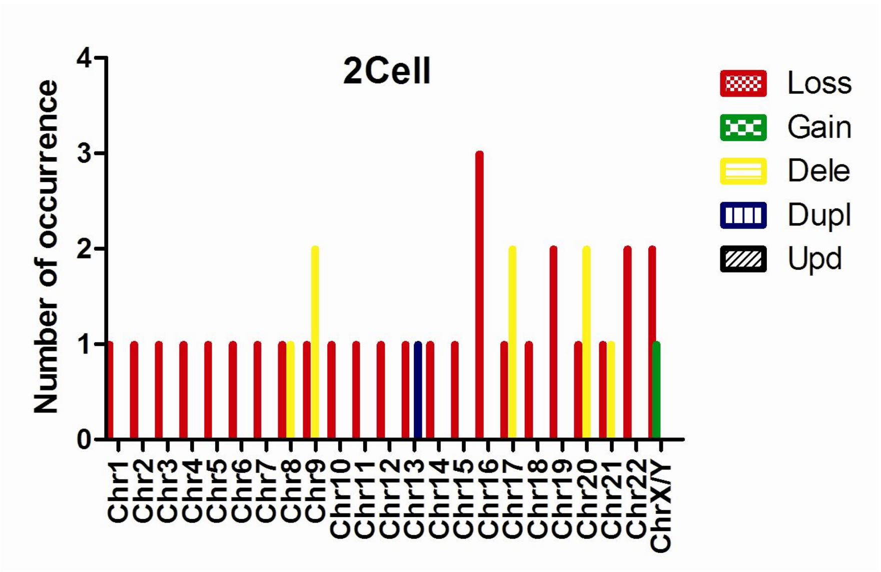

Supplementary Figure S11. Chromosome distribution of SNP array-based different abnormal signals from 2Cell-derived embryos

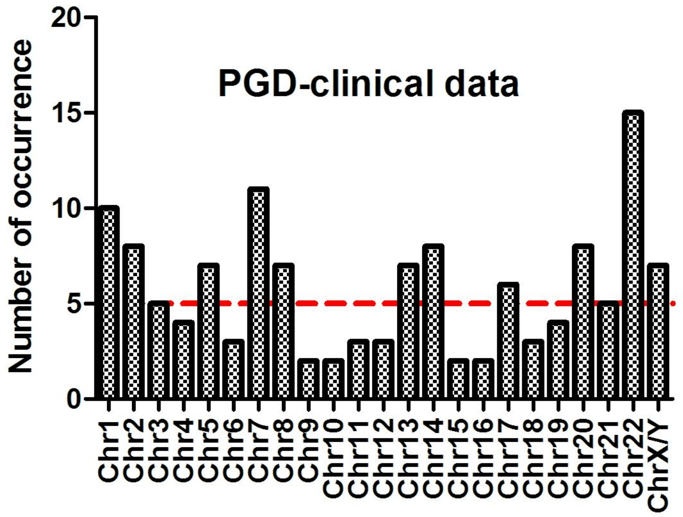

Supplementary Figure S12. Chromosome distribution of SNP array-based aberration signals from PGD-clinical data

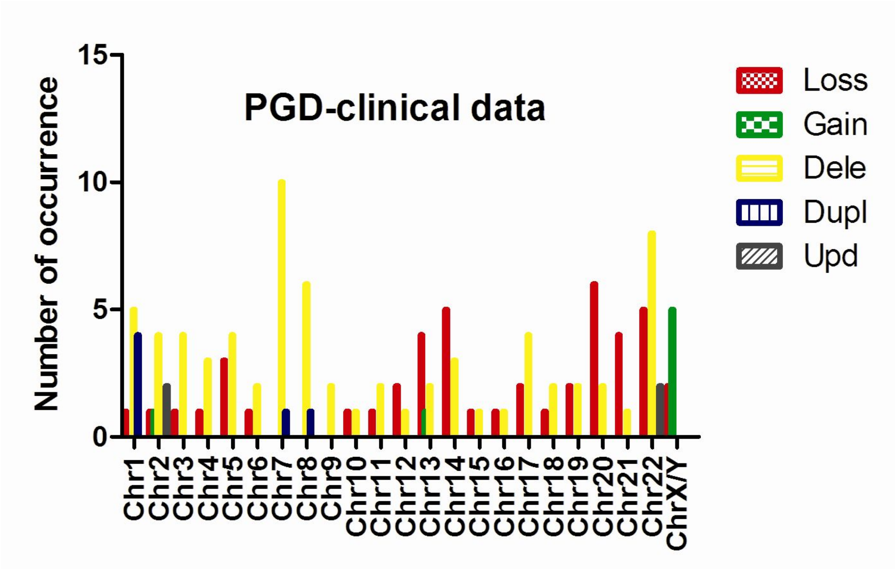

Supplementary Figure S13. Chromosome distribution of SNP array-based different abnormal signals from PGD-clinical data

Supplementary Table S1. Blastocyst development as a function of oocyte donor age

		Oocyte donor age (years) showing blastocyst development*												
	total§	21-22	23-24	25-26	27-28	29-30	31-32	33-34	35-36	37-38	39-40	41-42	43-44	45
0PN	90	1/1	1/4	4/17	1/8	2/11	2/18	1/13	3/8	0/6	0/1	0/3	-	-
1PN	28	0/4	0/5	1/5	0/2	0/5	0/1	0/1	0/2	0/1	-	0/1	0/1	-
2PN	385	2/8	12/42	15/82	8/44	13/59	14/50	9/36	6/24	3/15	1/7	4/18	-	-
3PN	893	1/16	5/74	14/128	9/115	21/126	20/122	9/107	8/88	6/68	3/16	3/23	1/10	
≥4PN	184	1/7	0/18	1/28	1/19	0/18	0/27	2/21	1/19	0/19	0/6	0/2	-	-
2-cell	69	1/1	3/8	13/16	3/5	12/15	6/10	3/3	2/5	1/2	-	0/3	0/1	-

*Number of embryos developing to blastocyst stage/total number of starting embryos in that age category

§Total embryo number at start.

Supplementary Table S2. Blastocyst development as a function of sperm donor age

		Sperm donor age showing blastocyst development*												
	total§	21-22	23-24	25-26	27-28	29-30	31-32	33-34	35-36	37-38	39-40	41-42	43-44	45
0PN	90	-	2/8	3/10	0/6	2/11	2/6	2/16	2/9	2/10	0/1	0/6	0/7	-
1PN	28	0/3	0/2	0/5	0/4	0/3	0/5	1/3	0/2	-	0/1	-	-	-
2PN	385	1/1	10/42	12/49	17/67	7/47	11/61	10/36	4/29	4/17	4/14	2/5	4/14	1/3
3PN	893	1/5	8/59	11/113	9/106	13/117	16/134	8/95	15/95	5/46	6/62	4/34	4/27	-
≥4PN	184	-	0/21	0/20	0/19	1/21	1/21	3/23	0/22	1/16	0/21	-	-	-
2-cell	69	-	2/6	14/19	1/2	9/14	6/6	2/2	9/15	1/1	-	0/4	-	-

*Number of embryos developing to blastocyst stage/total number of starting embryos in that age category

§Total embryo number at start.
